# Supplementary material for: Cytotoxic Potential of the Monoterpene Isoespintanol against Human Tumor Cell Lines
Source: Int J Mol Sci. 2024 Apr 23;25(9):4614. doi: 10.3390/ijms25094614 (PMC11083712; doi:10.3390/ijms25094614)
Supplement: Supplementary file 1 [file ijms-25-04614-s001.zip › ijms-2944963-supplementary.pdf]

International Journal of *Molecular Sciences*

MDPI

Article

Cytotoxic Potential of the Monoterpene Isoespintanol Against Human Tumor Cell Lines

Orfa Inés Contreras Martínez[1], Alberto Angulo Ortíz[2]*, Gilmar Santafé Patiño[2], Fillipe Vieira Rocha[3], Karine Zanotti[3], Dario Batista Fortaleza[3], Tamara Teixeira[3] and Jesús Sierra Martínez[4]

[1] Biology Department, Faculty of Basic Sciences, University of Córdoba, Montería 230002, Colombia.
[2] Chemistry Department, Faculty of Basic Sciences, University of Córdoba, Montería 230002, Colombia.
[3] Chemistry Department, Federal University of São Carlos, São Carlos, SP CEP 13565-905, Brazil.
[4] Genetics and Evolution Department, Federal University of São Carlos, São Carlos, 13565-905, SP, Brazil.

Supplementary Materials

Table S1. *IC_{50} values of cisplatin on MDA-MB-231, A549, DU145, A2780, A2780-cis and MRC5 monolayers.*

Cell lines	IC_{50} cisplatin (μM)
MDA-MB-231	2.40 ± 0.21
A549	14.40 ± 1.40
DU145	2.30 ± 0.42
A2780-cis	25.61 ± 0.29
A2780	11.17 ± 0.30
MRC5	12.47 ± 0.15
